# Mechanistic Characteristics of Surface Modified Organic Semiconductor g-C_3_N_4_ Nanotubes Alloyed with Titania

**DOI:** 10.3390/ma10010028

**Published:** 2017-01-03

**Authors:** Lan Ching Sim, Wei Han Tan, Kah Hon Leong, Mohammed J. K. Bashir, Pichiah Saravanan, Nur Atiqah Surib

**Affiliations:** 1 Department of Environmental Engineering, Faculty of Engineering and Green Technology, Universiti Tunku Abdul Rahman, Kampar 31900, Perak, Malaysia; weihan9316@1utar.my (W.H.T.); khleong@utar.edu.my (K.H.L.); jkbashir@utar.edu.my (M.J.K.B.); 2 Department of Environmental Science and Engineering, Indian Institute of Technology (ISM), Dhanbad 826004, Jharkhand, India; pichiahsaravanan@gmail.com; 3 Department of Environmental Engineering, Faculty of Engineering, Universiti Malaya, Kuala Lumpur 50603, Malaysia; nuratiqah1990@siswa.um.edu.my

**Keywords:** TiO_2_, g-C_3_N_4_, visible light, alkaline hydrothermal, Bisphenol A (BPA)

## Abstract

The visible-light-driven photocatalytic degradation of Bisphenol A (BPA) was investigated using the binary composite of alkaline treated g-C_3_N_4_ (HT-g-C_3_N_4_) deposited over commercial TiO_2_ (Evonik Degussa GmbH, Essen, Germany). The existence and contribution of both TiO_2_ and g-C_3_N_4_/HT-g-C_3_N_4_ in the composite was confirmed through various analytical techniques including powder X-ray diffraction (XRD), high-resolution transmission electron microscopy (HRTEM), field emission scanning electron microscopy (FESEM), Fourier transform infrared spectroscopy (FTIR), X-ray photoelectron spectroscopy (XPS), ultraviolet-visible diffuse reflectance spectra (UV-vis-DRS), and photoluminescence (PL) analysis. The results showed that the titania in the binary composite exhibited both pure rutile and anatase phases. The morphological analysis indicated that the spongy “morel-like” structure of g-C_3_N_4_ turned to nanotube form after alkaline hydrothermal treatment and thereby decreased the specific surface area of HT-g-C_3_N_4_. The low surface area of HT-g-C_3_N_4_ dominates its promising optical property and effective charge transfer, resulting in a deprived degradation efficiency of BPA two times lower than pure g-C_3_N_4_. The binary composite of HT-g-C_3_N_4_/TiO_2_ exhibited excellent degradation efficiency of BPA with 2.16 times higher than the pure HT-g-C_3_N_4_. The enhanced photocatalytic activity was mainly due to the promising optical band gap structure with heterojunction interface, favorable specific surface area, and good charge separation.

## 1. Introduction

Since the breakthrough discovery of photocatalytic splitting of water with titanium dioxide (TiO_2_) electrodes by Fujishima and Honda [1], TiO_2_ is widely used owing to its outstanding properties such as wide band gap, low cost, environmental-friendliness, non-toxicity, high photocatalytic capability, and high chemical stability [2,3]. Nevertheless, the high recombination of photoinduced electron-hole pairs and poor visible light response of TiO_2_ needs to be overcome to enhance the photocatalytic performance [4,5]. Among the heterojunction semiconductors, graphitic carbon nitride (g-C_3_N_4_) has been considered as one of the ideal candidates to alloy with TiO_2_ for photocatalytic application due to its high thermal stability, chemical stability, and visible absorption properties [6]. However, the fast charge recombination and poor conductivity of g-C_3_N_4_ are the main factors that have restricted its photocatalytic performance [7]. These limitations can be overcome through a modified structure of g-C_3_N_4_ to one-dimensional (1D) nanostructures (wires, tubes, rods, belts, fibers, etc.). They possess excellent properties like field emissions, gas sensing, photoconductivity, and phonon and electron transport properties since they possess a high surface to-volume ratio and more active sites [8].

Jin and co-workers [9] fabricated high specific surface area nanotube g-C_3_N_4_ via a simple two-step condensation method. Their findings showed 12 times higher photocatalytic activity than bulk g-C_3_N_4_ under visible light due to the higher surface area, the unique morphology, and the number of defects. It was also found that g-C_3_N_4_ nanofibers exhibited good electrochemical performance as electrodes for supercapacitors and excellent photocatalytic activity toward photodegradation of RhB because of the existence of nitrogen, a higher surface area, suitable band gap, and fewer textural structure defects [8]. Since the notable discovery mentioned above, limited studies have been reported on the fabrication of 1D nanostructured g-C_3_N_4_. Very recently, Hao and co-workers [10] synthesized g-C_3_N_4_/TiO_2_ heterojunction photocatalysts via a facile calcination method. They found that the fast recombination of electron-hole pairs slowed down because the close interface contact between g-C_3_N_4_/TiO_2_ resulting in enhanced visible light photocatalytic activity for the degradation of RhB. The binary composite of TiO_2_ and g-C_3_N_4_ nanofibers prepared by Wang and co-workers [5] displayed the best photocatalytic degradation on RhB (up to 99%) when the g-C_3_N_4_ content was 0.8 wt %. Though many studies have revealed the beneficial results of the TiO_2_/g-C_3_N_4_ binary composites there are still a few hurdles in using this composite. 

The researchers have adopted different methods to fabricate g-C_3_N_4_ 1D nanostructures like nanorods, nanofibers, nanobelts, nanotubes, and nanowires [8,11,12] by using hard templates or via introduction of acidic chemicals. Hard templating routes consume hazardous chemicals like hydrogen fluoride (HF) and aqueous ammonium bifluoride (NH_4_F_2_) used to dissolve silica hard templates [13]. Therefore, hydrothermal technique has received considerable attention to synthesize tube-like nanostructure due to the simple apparatus set-up and milder reaction condition. In this study, g-C_3_N_4_ was prepared with the simple pyrolysis of urea while g-C_3_N_4_ nanotubes were achieved through one-step hydrothermal method. Urea was used as the precursor owing to its low cost, non-toxic nature, and also its molecular activity under thermal treatment [14]. The synthesized g-C_3_N_4_ nanotubes were combined with the Aeroxide^®^ P25 Degussa TiO_2_ (Evonik Degussa GmbH, Essen, Germany). The visible light driven photocatalysis of g-C_3_N_4_ was evaluated by degrading organic pollutant Bisphenol A (BPA).

## 2. Material and Methods

### 2.1. Preparation of g-C_3_N_4_ and HT-g-C_3_N_4_

Urea (R&M Chemicals, Essex, UK) was used as a precursor to synthesize graphitic carbon nitride (g-C_3_N_4_) through thermal heating method. A total of 10 g of urea was prepared in a crucible with a lid and dried in an oven at 80 °C for one day. The urea was then put in a muffle furnace operated under air atmosphere to heat up to 500 °C for 3 h, a yellowish product was obtained after this process. The yellowish product was washed with nitric acid (0.1 M) several times and distilled water to remove any residual alkaline species adsorbed on the sample surface. Then, 0.4 g of the sample was dried at 80 °C and several batches were combined to obtain 1.0 g portion of g-C_3_N_4_. The obtained g-C_3_N_4_ was well grounded in an agate mortar before alkaline hydrothermal treatment. The obtained g-C_3_N_4_ was mixed with 90 cm^3^ of NaOH (0.10 mol·dm^−3^) solutions in a pressure-tight Teflon-lined autoclave and was subjected to hydrothermal treatment at 150 °C for 18 h. After cooling down to room temperature, the solid product was dried at 80 °C for 24 h. The sample obtained in this treatment was denoted as HT-g-C_3_N_4_.

### 2.2. Synthesis of g-C_3_N_4_/HT-g-C_3_N_4_ hybridized TiO_2_

A 0.012 g sheet of HT-g-C_3_N_4_ was well dispersed in distilled water ultrasonically. Then, 0.4 g of Aeroxide^®^ P25 Degussa TiO_2_ (Evonik Degussa GmbH, Essen, Germany) was added to the solution and subjected to 70 °C for 1 h. The resulting suspension was then centrifuged and washed repeatedly with distilled water a few times and dried overnight at 60 °C. The sample obtained was denoted as HT-g-C_3_N_4_/TiO_2_. To prepare g-C_3_N_4_/TiO_2_, a similar synthesis route was repeated by replacing HT-g-C_3_N_4_ with g-C_3_N_4_.

### 2.3. Characterization

The powder X-ray diffraction (XRD, PANalytical-Empyrean, Almelo, The Netherlands) patterns were acquired with Cu Kα radiation at a scanning speed of 0.02 s^−1^. The morphology structures of the samples were observed on a field emission scanning electron microscope (FESEM, JSM-6701F, JEOL Ltd., Tokyo, Japan) at 20 kV. The lattice fringe images were dissected by high-resolution transmission electron microscopy (HRTEM, FEI-TECNAI F20, Hillsboro, OR, USA) using an accelerating voltage of 200 kV. Fourier transform infrared (FT-IR, Perkin Elmer Spectrum 400 spectrophotometer, Perkin Elmer, Wokingham, UK) spectra were conducted with the samples dispersed in KBr desiccative in the range of 400–4000 cm^−1^. The Brunauer–Emmett–Teller specific surface area and pore volume of samples were determined at liquid nitrogen temperature (77 K) based on nitrogen adsorption-desorption isotherms with TriStar II 3020 (Micrometrics^®^, Norcross, GA, USA). Ultraviolet-visible diffuse reflectance spectra (UV-vis-DRS) were obtained using Shimadzu UV-2600 spectrophotometer equipped with integrating sphere attachment with BaSO_4_ as a reference. Both Raman and photoluminescence (PL) spectra were acquired by using a Renishaw inVia Raman Microscope (Renishaw, Wotton-under-Edge, UK) with the excitation wavelength at 514 nm and 325 nm, respectively. The surface chemical composition of the samples was analyzed by X-ray photoelectron spectroscopy (PHI Quantera II, Ulvac-PHI, Inc., Kanagawa, Japan) with an Al K_α_ radiation source.

### 2.4. Photocatalytic Degradation of Organic Pollutants

The visible light photocatalysis of the synthesized samples was evaluated based upon the removal of BPA. The amount of the photocatalyst used in this experiment was 0.02 g. The prepared photocatalysts were immersed in a glass beaker containing 250 mL aqueous solutions for BPA (5 mg·L^−1^). Prior to photocatalysis, an adsorption-desorption equilibrium was established in the dark for 1 h. A 500 W tungsten-halogen lamp with a high-pass UV filter (λ < 420 nm) (SCF-50S-42L, OptoSigma, Tokyo, Japan) was used as visible light source. The degraded products were collected at regular intervals, then analyzed for residual BPA concentration using a liquid chromatography (Acquity UPLC H-Class, Waters, Milford, MA, USA) attached with C18 column (2.1 mm × 50 mm and 1.7 μm) at a detection wavelength of 226 nm. The mobile phase was water and acetonitrile (ACN) at a ratio of 60:40 with a flow rate of 0.4 mL·min^−1^. The photocatalytic experiments were carried out for 3.5 h.

## 3. Results and Discussion

### 3.1. FESEM and HRTEM

**Figure 1:**
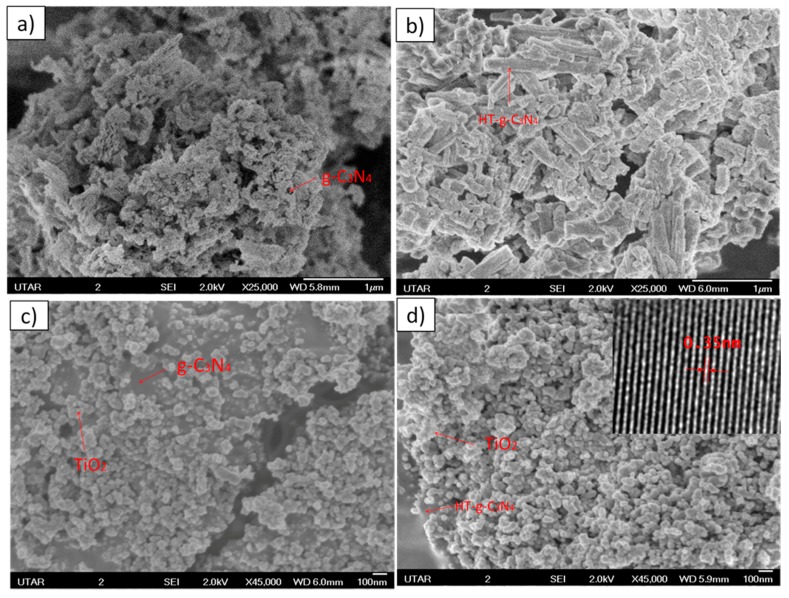
Field emission scanning electron microscopy (FESEM) images of (**a**) g-C_3_N_4_; (**b**) HT-g-C_3_N_4_; (**c**) g-C_3_N_4_/TiO_2_; (**d**) HT-g-C_3_N_4_/TiO_2_. The inset shows the lattice fringes of HT-g-C_3_N_4_/TiO_2_.

The surface of morphology of g-C_3_N_4_ before and after the hydrothermal treatment is shown in Figure 1. The spongy “morel-like” structure in Figure 1a reveals that the synthesized g-C_3_N_4_ possesses a high specific surface area. The alkaline hydrothermal treatment transformed the porous nanostructured of g-C_3_N_4_ to clustered nanotubes geometry with lower specific surface area (Figure 1b). This phenomenon is attributed to the complication of self-assembly process during the fabrication of 1D g-C_3_N_4_ nanotubes [11]. The alloyed TiO_2_ nanoparticles are well-distributed on the surface of porous structred g-C_3_N_4_ (Figure 1c). As illustrated in Figure 1d, the reducing specific surface area of HT-g-C_3_N_4_ hindered the uniform dispersion of TiO_2_ nanoparticles on their surface, resulting in the agglomeration of TiO_2_ onto HT-g-C_3_N_4_/TiO_2_. The inset in Figure 1d depicts the lattice fringes that signify the presence of TiO_2_ (0.35 nm) in the prepared binary composite. 

### 3.2. XRD and BET

**Figure 2:**
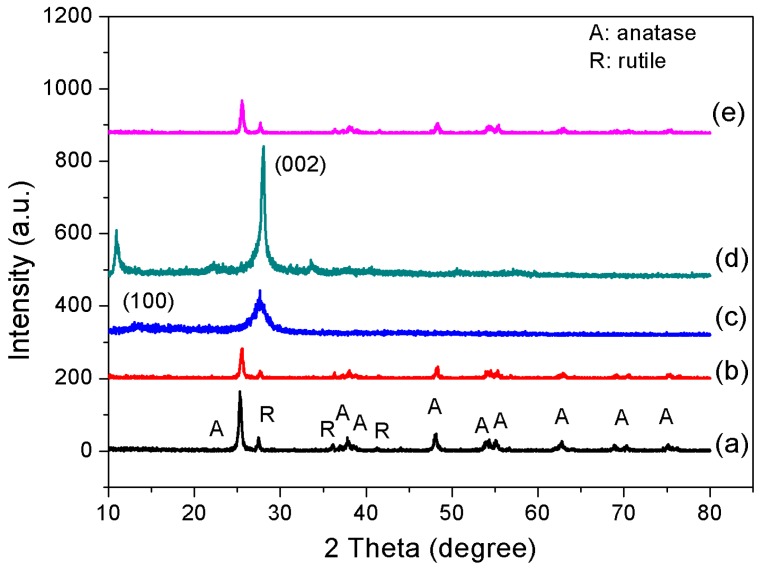
X-ray diffraction pattern of photocatalysts (**a**: P25; **b**: HT-g-C_3_N_4_/TiO_2_; **c**: g-C_3_N_4_; **d**: HT-g-C_3_N_4_; **e**: g-C_3_N_4_/TiO_2_).

Figure 2 shows XRD pattern of various synthesized samples. A weak (1 0 0) diffraction peak at 13.1° was observed for the pure g-C_3_N_4_, indicating the periodic structure of intra-planar tri-s-triazine packing [15]. The strong (0 0 2) peak at 27.4° signifies the interlayer stacking reflection of conjugated aromatic systems [16]. The intensity of (1 0 0) diffraction peak of HT-g-C_3_N_4_ increases and shifts toward the lower diffraction angle at 10.8°. This implies that the alkaline hydrothermal treatment of g-C_3_N_4_ stretched out the intra-planar separation of ordered tri-s-triazine packing [17,18]. It is observed that the intensity of two distinct diffraction peaks becomes lower for the binary composites because of the low amount of loading on the surface of the composites [19]. The two obvious peaks of the tetragonal TiO_2_ anatase phase (JCPDS No. 21-1272) appeared at 25.3° (1 0 1) and 48.0° (2 0 0). While the peaks at 27.4°, 36.1°, and 41.2° were ascribed to (1 1 0), (1 0 1), and (1 1 0) planes of rutile TiO_2_ (JCPDS No. 21-1276), respectively. The surface characteristics of the samples including binary composites obtained through Barret–Joyner–Halender (BJH) method are summarized in Table 1. The alkaline hydrothermal treatment brought a significant modification on its (g-C_3_N_4_) surface, whereby the specific surface area of g-C_3_N_4_ was reduced from 71.8 to 6.3 m^2^·g^−1^. The nucleation effect thus led to drastic changes by increasing pore size (~236.6 nm) and crumpling some pores partially, resulting in a diminished total pore volume and BET surface area, respectively [20]. However, the surface area of HT-g-C_3_N_4_/TiO_2_ is found to be much higher (~53.1 m^2^·g^−1^) as compared to that of HT-g-C_3_N_4_. The enhancement in surface area is attributed to the change in morel-like morphology to nanotubes, which suppressed the entry of TiO_2_ nanoparticles into the HT-g-C_3_N_4_ with lower pore volume. Therefore, the aggregation of TiO_2_ occurred only on the external surface of HT-g-C_3_N_4_ without clogging the pores of nanostructures. 

### 3.3. UV-DRS

**Figure 3:**
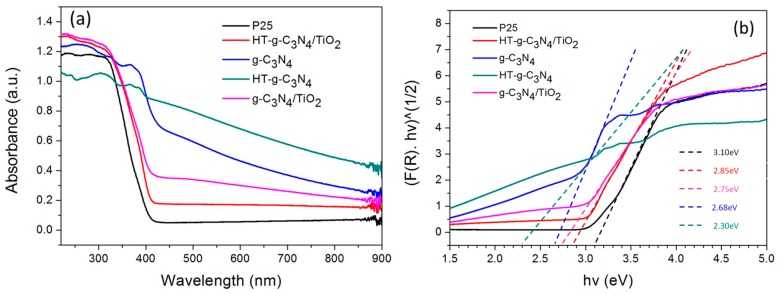
(**a**) Ultraviolet-visible (UV-vis) absorption spectra; (**b**) Tauc plots of P25, HT-g-C_3_N_4_/TiO_2_, g-C_3_N_4_, HT-g-C_3_N_4_, and g-C_3_N_4_/TiO_2_.

Figure 3a displays the visible light harvesting capability of the samples with the following sequence; HT-g-C_3_N_4_ > g-C_3_N_4_ > g-C_3_N_4_/TiO_2_ > HT-g-C_3_N_4_/TiO_2_ > P25. Among the samples, both virgin and HT-g-C_3_N_4_ exhibited a significant red shift and thus the introduction of them onto the surface of TiO_2_ greatly stimulated the visible light absorption with an apparent shift at 450 nm in the binary composites. Moreover, the alkaline hydrothermal treatment is foreseen as an effective approach to promote visible-light absorption of g-C_3_N_4_ owing to the increase in the scattering factor originating from the diminished porous structure of HT-g-C_3_N_4_ [21]. The Tauc plots in Figure 3b show the band gap of the studied samples. By plotting (*F*(*R*)*hν*)^1/2^ against *hν*, the band gap of each sample can be obtained, where Kubelka-Munck function *F*(*R*) is derived from equation as below:

*F*(*R*) = (1 − *R*)^2^/2*R* (1)

where *R* is the diffuse reflectance and *hν* is the photon energy. HT-g-C_3_N_4_ was the optimum sample according to the calculated band gap energy. It demonstrates a strong harvesting ability in the visible light spectrum with a band edge at 531 nm corresponding to a band gap of 2.30 eV. A more perfect packing, electronic-coupling, and quantum confinement effect that shifts conduction and valence band edges could also be a factor that contributes to this phenomenon [8].

### 3.4. FTIR and PL Spectra

**Figure 4:**
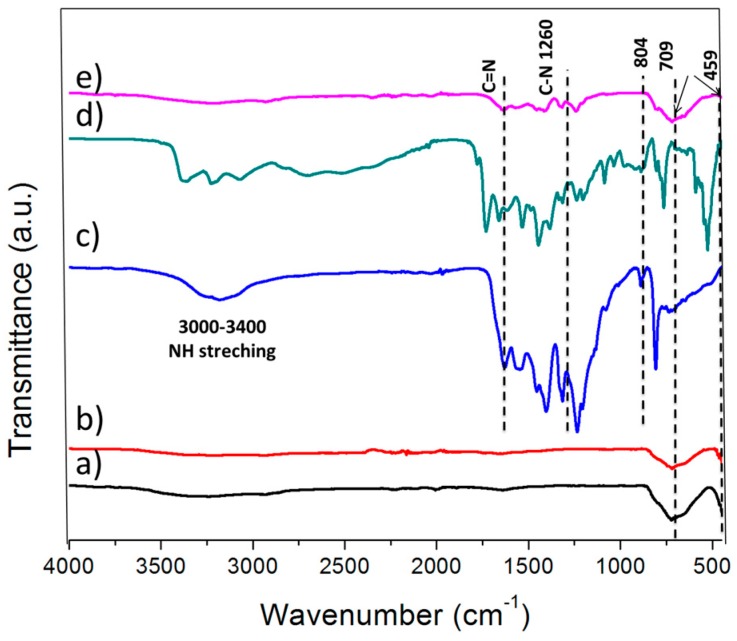
Absorption and emission Fourier transform infrared spectra of (**a**: P25; **b**: HT-g-C_3_N_4_/TiO_2_; **c**: g-C_3_N_4_; **d**: HT-g-C_3_N_4_; **e**: g-C_3_N_4_/TiO_2_).

Figure 4 shows the Ti–O–Ti and Ti–O stretching vibration modes in anatase crystals was assigned by P25 due to its main peak being in the range of 500–800 cm^−1^. For the g-C_3_N_4_, the N–H stretching was found at the broad peak from 3000 to 3400 cm^−1^ [22]. The peaks ranging between 1200 and 1640 cm^−1^ were attributed to the presence of two major bonds in g-C_3_N_4_. The sp^2^ C=N stretching vibration modes were assigned to the peak at 1630 cm^−1^, while the other peaks at the range of 1200–1640 cm^−1^ were assigned to the aromatic sp^3^ C–N bonds [10]. The sharp peak at 804 cm^−1^ resembled the s-triazine ring vibrations [23]. The spectrum of g-C_3_N_4_ was similar to g-C_3_N_4_/TiO_2_ and HT-g-C_3_N_4_ since both of them mainly consist of g-C_3_N_4_.

**Figure 5:**
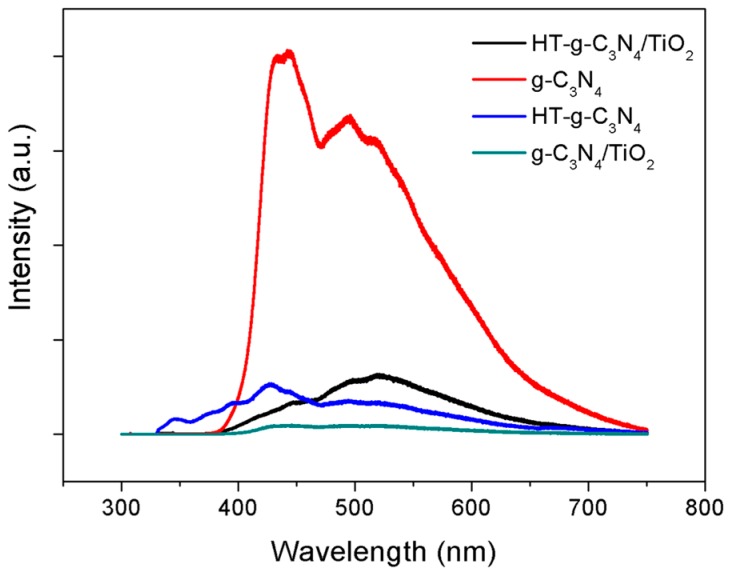
Photoluminescence spectra of P25, HT-g-C_3_N_4_/TiO_2_, g-C_3_N_4_, HT-g-C_3_N_4_, and g-C_3_N_4_/TiO_2_.

The PL spectra in Figure 5 were obtained to understand the separation of charge carrier progressed in photocatalysis for all samples. The emission peak of g-C_3_N_4_ is the highest compared to the rest, implying the rapid recombination of photogenerated electrons and holes. The defects in crystal structure of g-C_3_N_4_ become the recombination centers for photoinduced electrons and holes during the photocatalysis [24]. However, the emission peak was obviously quenched after alloying TiO_2_ with g-C_3_N_4_, the lifespan of the electrons and holes was extended when the electrons mobilize from g-C_3_N_4_ to the conduction band of TiO_2_ [25]. The PL intensity of HT-g-C_3_N_4_ is also lower than that of g-C_3_N_4_, signifying a sharp decline in the number of defects achieved through alkaline hydrothermal treatment. Further, it also indicates that the 1D nanotube structure of HT-g-C_3_N_4_ offers sufficient lengths to capture incident photons and provides facile separation of charges and results in higher photoefficiency.

### 3.5. XPS Analysis

**Figure 6:**
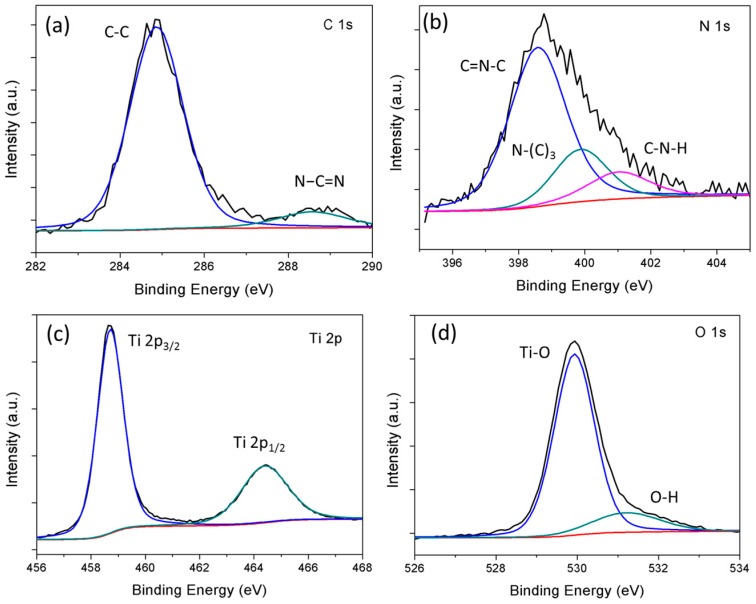
Core level XPS spectra of (**a**) C 1s; (**b**) N 1s; (**c**) Ti 2p; and (**d**) O 1s of binary composite.

The chemical states of C, N, Ti, and O in binary composites are investigated by XPS and the obtained results are displayed in Figure 6. The C 1s spectrums are deconvoluted into two distinct peaks with binding energies at 284.8 eV and 288.3 eV, attributable to the C–C coordination of sp^2^ graphitic carbon [26,27] and sp^2^-bonded carbon (N–C=N) of the s-triazine rings, respectively [28]. For the N 1s spectrum, the peak at 398.6 eV is assigned to sp^2^ hybridized aromatic N bonded to carbon atoms (C=N–C). The peak at 399.7 eV confirms the presence of s tertiary nitrogen N–(C)_3_ group linking structural motif (C_6_N_7_) or amino groups carrying hydrogen ((C)_2_–N–H) in connection with structural defects and incomplete condensation [29]. Another peak at 401.1 eV is attributable to the quaternary N bonded three carbon atoms (C–N–H) in the aromatic cycles [14,30,31]. The two distinct peaks observed at 459 eV (Ti 2p_3/2_) and 464.5 eV (Ti 2p_1/2_), both correspond to Ti^4+^ in pure anatase [32]. The O 1s spectrum displays two peaks at 530 eV and 531.3 eV which correspond to Ti–O bond and O–H bond, respectively [33]. 

### 3.6. Photocatalytic Performance

**Figure 7:**
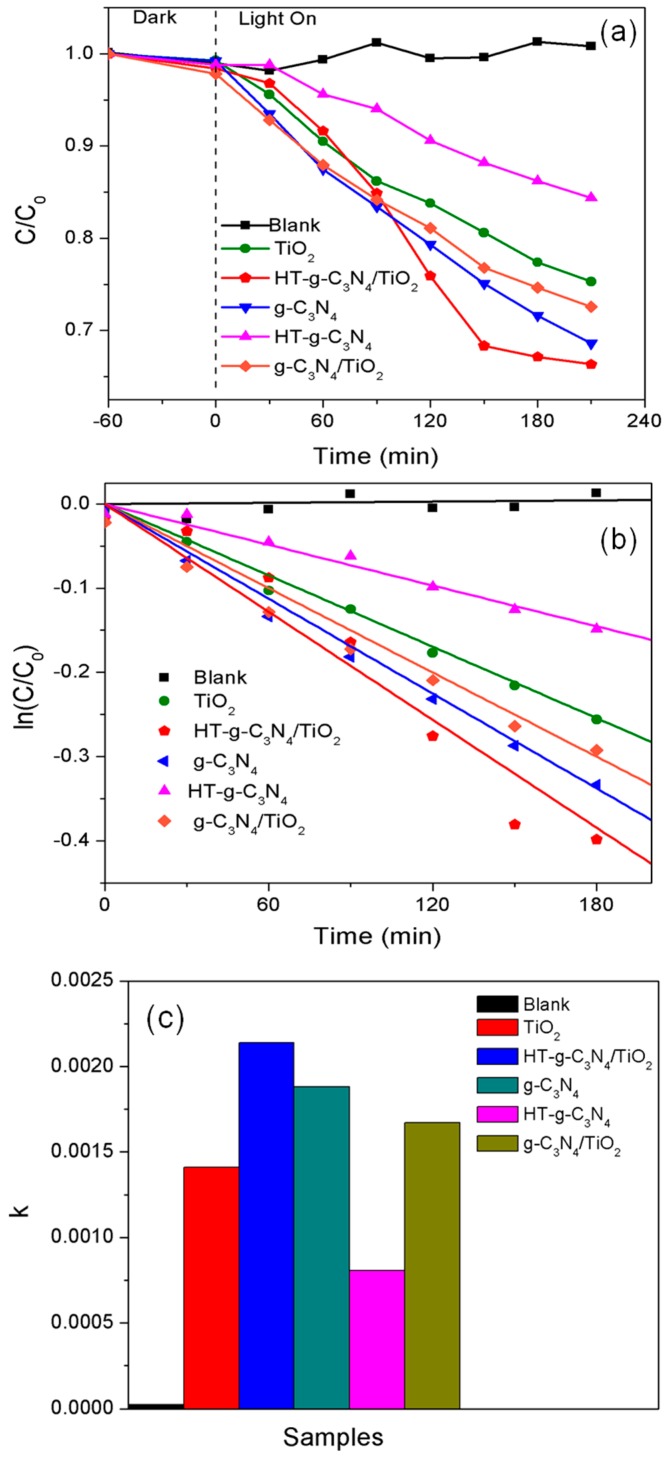
(**a**) Photocatalytic degradation of Bisphenol A (BPA) as a function of reaction time; (**b**) Fitted first order kinetic plots for BPA degradation; and (**c**) Apparent rate constant *k*^−1^.

Figure 7a depicts visible-light-induced photocatalysis reaction of prepared photocatalysts. The observed degradation data were fitted to the simple kinetic model in Figure 7b,c. The first-order reaction kinetics are expressed by equation:

ln(*C*/*C*_0_) = −*kt* (2)

where *k* is the first-order reaction constant, *C*_0_ and *C* are the BPA concentrations in the solution at times 0 and *t*, respectively.

The photocatalytic performance followed an order of HT-g-C_3_N_4_/TiO_2_ > g-C_3_N_4_ > g-C_3_N_4_/TiO_2_ > TiO_2_ > HT-g-C_3_N_4_ > blank. There was almost no change with time in the absence of catalyst, proving that BPA is a poor photosensitizing compound. All samples showed a relatively slight adsorption capacity (~1%) towards the BPA during the dark adsorption process. The P25 TiO_2_ showed a relatively good photocatalytic degradation of BPA due to the positive interaction between anatase and rutile phase which facilitated the charge separation. Although HT-g-C_3_N_4_ possesses 1D nanotube structure and superior visible light harvesting properties, it did not lead to a greater photoefficiency. Its photocatalytic performance was restricted by its smaller specific surface area (6.29 m^2^·g^−1^) compared to that of pure g-C_3_N_4_ (71.78 m^2^·g^−1^) after the change in morphology. However, it was clear that the loading of TiO_2_ onto the surface of HT-g-C_3_N_4_ significantly increased the surface area and improved the degradation efficiency of BPA at 2.16 times higher than the pure HT-g-C_3_N_4_. The rapid transportation of photoinduced charge carriers at the interface between HT-g-C_3_N_4_ and TiO_2_ is due to the heterostructure of the binary composite which also played a vital role in the enhancement of photocatalytic performance [34,35]. Although g-C_3_N_4_ g suffered from the fast carrier recombination rate [16], it still exhibited better degradation efficiency of BPA (*k* = 0.00188 min^−1^) when compared to that of HT-g-C_3_N_4_ (*k* = 0.00081 min^−1^). This is attributed to its excellent visible light harvesting properties and relatively large surface area, and thus more active adsorption sites were available for the reactants. In the binary composite of g-C_3_N_4_/TiO_2_, the loading of TiO_2_ nanoparticles significantly suppressed the carrier recombination rate but decreased the surface area of the binary composite, leading to a degradation efficiency of g-C_3_N_4_/TiO_2_ 1.24 times lower than HT-g-C_3_N_4_/TiO_2_.

**Figure 8:**
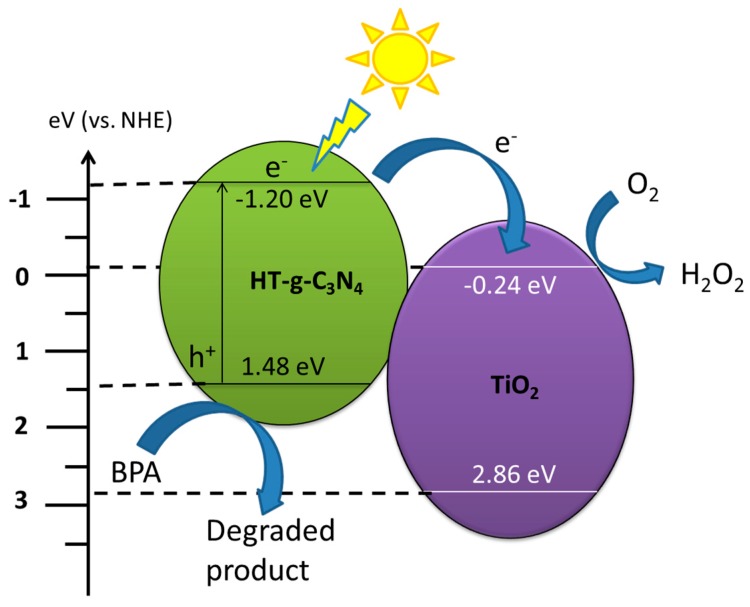
Schematic diagram of electron transfer and degradation mechanisms of BPA.

The degradation mechanism in Figure 8 displays that HT-g-C_3_N_4_ was excited by artificial visible light (λ > 420 nm) and generated electron and hole pairs. The edge potential of conduction band (CB) and valence band (VB) of a semiconductor at the point of zero charge was estimated according to the following equations:

*E*_VB_ = *X − E*_C_ + 0.5*E*_g_ (3)

*E*_CB_ = *E*_VB_* − E*_g_ (4)

where *X* is the electronegativity of the semiconductor; *E*_VB_ and *E*_CB_ are the valence band and conduction band edge potential, respectively; *E*_C_ is the energy of free electrons on the hydrogen scale (~4.5 eV vs. NHE); and *E*_g_ is the band gap energy of the semiconductor. The *X* values of the HT-g-C_3_N_4_ and TiO_2_ are 4.64 eV and 5.81 eV, respectively [36,37]. The band gap energy of HT-g-C_3_N_4_ and TiO_2_ are 2.68 eV and 3.1 eV, respectively. The VB and CB were theoretically calculated at (1.48 eV, −1.20 eV) and (2.86 eV, −0.24 eV) for HT-g-C_3_N_4_ and TiO_2_, respectively. The narrow band gap energy (2.68 eV) of HT-g-C_3_N_4_ enables easy excitation of electrons upon the irradiation of visible light. The photoinduced electrons transferred from the valence band (VB) to the conduction band (CB) of HT-g-C_3_N_4_. Although there was no excitation in TiO_2_, it can accumulate the electrons injected from the CB of HT-g-C_3_N_4_ since the CB edge potential of HT-g-C_3_N_4_ (−1.20 eV) is more negative than that of TiO_2_ (−0.24 eV). It is noteworthy that the dominant negative redox potential of O_2_/•O_2_^−^ (−0.33 eV) inhibits the reduction reaction between the trapped electrons in the CB of TiO_2_ and O_2_. However, these electrons can reduce O_2_ to H_2_O_2_ and further to hydroxyl radical (•OH) due to the favorable redox potential of O_2_/H_2_O_2_ (0.695 eV) [19,38]. The generated strong oxidizing radicals (standard redox potential +2.8 eV) were actively involved in the degradation of BPA. Meanwhile, the photoinduced holes in HT-g-C_3_N_4_ with higher oxidation potential (1.48 eV vs. NHE) can directly oxidize BPA because the VB level of HT-g-C_3_N_4_ (1.48 eV) is too low to oxidize H_2_O (2.27 eV) [39]. In the binary composite, the loading of TiO_2_ onto the surface of HT-g-C_3_N_4_ could act as an electron acceptor to facilitate the separation of electron-hole pairs and store the separated electrons. Hence, the lifetime of charge carries was prolonged, leading to improved photocatalytic performance. In the photocatalytic degradation of BPA, assorted intermediates like benzoquinone, hydroxyacetophenon, phenol, 2-(4-hydroxyphenyl)-2-propanol, and isopropylphenol have been determined by several researchers [40,41]. Besides, short-chain aliphatic acids such as citric, maleic, acetic, tartaric, and formic acids ensuing from aromatic cleavage were also reported [40].

## 4. Conclusions

The binary composites, HT-g-C_3_N_4_/TiO_2_ and g-C_3_N_4_/TiO_2_, were successfully synthesized via a facile method. The incorporation of both HT-g-C_3_N_4_ and g-C_3_N_4_ significantly shifted the light absorption towards the visible region. The excellent electron and hole separation in the resulting binary composites was reflected in the PL spectra. The morphology change from porous to nanotube structure after alkaline hydrothermal treatment contributed to a trivial photocatalytic activity of HT-g-C_3_N_4_. It was overcome by the deposition of TiO_2_ onto the surface of HT-g-C_3_N_4_ which increased the specific surface area in binary composite, leading to enhanced photocatalytic activity. The presence of TiO_2_ in the binary composite also served as an electron acceptor which rendered oriented transfer of the charge carriers across the heterojunction interface. This simple illustrated methodology for the design of functional photocatalysts with tailored phenomenon can drive other reaction pathways with environmental applications sustainably. The enhanced catalytic efficiency is attributed predominantly to the narrow band gap structure with a heterojunction interface and prolonged lifetime of charge carriers.

## Figures and Tables

**Table 1:** S_BET_, total pore volume and pore size of P25, HT-g-C_3_N_4_/TiO_2_, g-C_3_N_4_, HT-g-C_3_N_4_, and g-C_3_N_4_/TiO_2_.

Sample	S_BET_ (m^2^·g^−1^)	Total Pore Volume (cm^3^·g^−1^)	Pore Size (nm)
P25	52.8	0.155	117.5
HT-g-C_3_N_4_/TiO_2_	53.1	0.357	269.0
g-C_3_N_4_	71.8	0.299	166.6
HT-g-C_3_N_4_	6.3	0.037	236.6
g-C_3_N_4_/TiO_2_	45.3	0.270	242.3
